# Safety and location analysis of transumbilical endoscopic submucosal dissection with single-basin lymph node dissection in the upper gastric body: a porcine model

**DOI:** 10.1007/s00464-019-06801-2

**Published:** 2019-04-23

**Authors:** Sang-Ho Jeong, Jae-Seok Min, Ji-ho Park, Soon-Chan Hong, Eun-Jung Jung, Young-tae Ju, Chi-Young Jeong, Han Shin Lee, Miyeong Park, Young-Joon Lee, Chang Yoon Ha

**Affiliations:** 1 Department of Surgery, Gyeongsang National University School of Medicine, Gyeongsang National University Hospital, 15, Jinju-daero 816 beon-gil, Jinju, Gyeongsangnam-do 52727 South Korea; 2 Gyeongsang National University Changwon Hospital, Changwon, South Korea; 3 Department of Surgery, Dongnam Institute of Radiological and Medical Sciences, Cancer Center, Busan, South Korea; 4 Department of Anesthesiology, Gyeongsang National University School of Medicine, Jinju, South Korea; 5 Department of Internal Medicine, Gyeongsang National University School of Medicine, Jinju, South Korea

**Keywords:** Endoscopic submucosal dissection, Single-port surgery, Gastric neoplasm, Endoscope, Laparoscopy, Porcine

## Abstract

**Background:**

In our previous study, transumbilical endoscopic submucosal dissection (TU-ESD) was revealed to be feasible, but delayed gastric perforation was observed in 30% of ESD sites. In this study, we aimed to verify locations at which it is feasible to perform TU-ESD in the upper gastric body and to demonstrate the safety of TU-ESD in single-basin lymph node dissection (SBLND).

**Methods:**

In vitro, TU-ESD was performed at three lesion sites (anterior wall, AW; posterior wall, PW; and lesser curvature, LC) in each porcine stomach using an EASIE-R tray (cases = 10). In vivo, TU-ESD was performed with SBLND in 9 pigs. Seven days after the operation, the pigs were sacrificed and examined.

**Results:**

In the in vitro feasibility study, the TU-ESD time was significantly faster in the PW group (5.9 ± 2.0 min) than in the LC group (8.5 ± 1.5 min) (*p *< 0.05) in all 10 cases. In the in vivo survival study, TU-ESD with SBLND was successfully performed without any complications (*N* = 9). There were no cases of delayed perforation, and healing ulcers were found in all pigs 7 days after the operation. Ulcer size (5.2 ± 3.5 cm^2^) was approximately 36% smaller than that observed at the ESD operation site (8.1 ± 1.9 cm^2^) (*p *= 0.05). Epithelialization in the margin and healing of the gastric ulcers were confirmed by microscopy.

**Conclusions:**

TU-ESD with SBLND is a feasible and safe method. The upper posterior gastric body could be the most feasible location for performing TU-ESD, perhaps because of the difference in the subcutaneous dissection time.

Although the incidence and mortality rates of gastric cancer have gradually decreased in northeast Asia, gastric cancer remains the fifth most common malignancy and the third leading cause of cancer-related deaths worldwide [1, 2]. The prevalence of early gastric cancer (EGC) is higher than 50% in northeast Asia because of the recently implemented screening system and advances in endoscopic diagnosis [3]. The most common site of gastric cancer is the lower one-third of the stomach, in which over half of all such cases occur in Korea; however, the proportion of recent cases of gastric cancer occurring in the upper one-third of the stomach steadily increased from 11% in 1995 to 16% in 2014 [4]. For the removal of EGC (cT1a), endoscopic procedures, such as endoscopic submucosal dissection (ESD), have been recognized as optimal minimally invasive procedures.

In ECG, the tumor location is one of the most significant factors for achieving a safe and complete resection. Lesions of the posterior wall (PW) and upper third of the gastric body are highly associated with incomplete resection and fatal complications, such as perforation, probably because of the technical difficulty of the procedure [5]. Additionally, the ESD procedure can induce delayed gastric emptying, especially in cases of EGC located in the upper third of the lesser curvature (LC) [6]. To avoid these adverse outcomes after ESD, proximal or total gastrectomy is usually performed instead of ESD when the upper one-third of the gastric body is involved.

Except for patients with EGC who can be treated by ESD, in theory, EGC patients should undergo radical gastrectomy even if diagnosed at an early stage [7]. Currently, the novel treatment is sentinel basin dissection, which is sometimes referred to as minimally focused lymphadenectomy with concurrent minimal resection of the stomach and includes wedge resection, segmental resection, and intraoperative ESD; this procedure has been recommended for ECG patients with nonmetastatic sentinel lymph nodes (LNs) [8]. Recently, several studies reported performing endoscopic submucosal dissection with laparoscopic sentinel LN dissection [9–13]. Additionally, a large-scale multicenter trial (SENORITA) is underway in the Republic of Korea, and the results will be presented in the future [14, 15]. In our previous study, transumbilical ESD (TU-ESD) with single-port laparoscopic lymph node dissection (L-LND) was found to be feasible and outperformed transoral ESD with multiport L-LND in upper one-third gastric tumors in terms of the complication rate [16]. However, delayed gastric perforation was observed in 30% of the ESD sites (3/10), and we believe that a lack of blood circulation is one possible cause of this complication. We therefore concluded that one-sided basin dissection would be safer than two-sided dissection; however, we did not confirm that one-sided L-LND was safer than two-sided L-LND or evaluate different outcomes of ESD with regard for the circumferential location of upper EGC tumors.

The aims of this study were to verify the feasibility of performing TU-ESD in the upper gastric body at anterior wall (AW), PW, and LC sites and demonstrate the safety of TU-ESD with single-basin lymph node dissection (SBLND).

## Materials and methods

### 1. In vitro feasibility study of TU-ESD by location

#### Step 1: Preparation of the in vitro model

We performed an in vitro study using an EASIE-R tray (ENDOSIM, Hudson, MA, USA) with porcine stomach specimens obtained from a local slaughterhouse (SK industry, Jinju, Republic of Korea).

#### Step 2: Creation of the TU route in an in vitro model

**Fig. 1:**
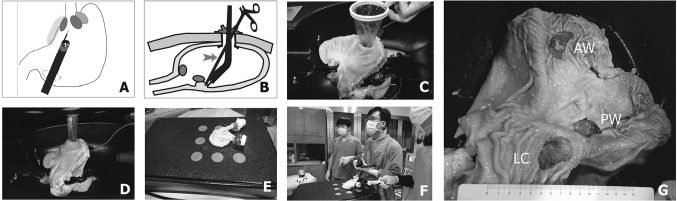
In vitro feasibility study of TU-ESD by circumferential location in the upper body of the porcine stomach. Schematic figure of the transumbilical route used in the in vitro model with an EASIE-R tray system (**A**, **B**). We made an incision via gastrostomy, and we then inserted the wound protector through the gastrostomy site (**C**, **D**). The stomach was then insufflated with CO_2_ through a hand-made glove port with a trocar (**E**). We dissected the connective tissue of the submucosa with a hook knife and retracted the specimen using a laparoscopic grasper that was inserted through the assist port (red arrow, **B**, **F**). After the procedure, the operation times, tissue weights, and specimen diameters were recorded (**G**)

We created a TU in vitro model using an EASIE-R tray system (Fig. 1A, B). We made an incision at the selected gastrostomy site, which corresponded to the greater curvature side of the antrum AW for humans (Fig. 1C). We inserted a wound protector through the gastrostomy site and insufflated the stomach with CO_2_ (6 mmHg of CO_2_ pressure) through the handmade glove port (Fig. 1D, E).

#### Step 3. TU-ESD in an in vitro model

Endoscopic submucosal dissection was performed at three different sites (AW, PW and LC) in each porcine stomach (*n* = 10) by an expert endoscopist (Dr. Ha) using white-light endoscopy and chromoendoscopy with an indigo carmine solution (GIF-H260; Olympus Optical Co., Ltd., Tokyo, Japan). Marks were made around the lesions with a dual knife, and the mucosa surrounding the lesion was precut. The connective tissue of the submucosa was dissected with a hook knife, and the specimen was retracted using a laparoscopic grasper (Endo Grasp, 5 mm; US Surgical, Norwalk, CT, USA) that was inserted through the assist port (red arrow, Fig. 1B, F). The operation time, tissue weight, and specimen diameter were recorded. The long and short diameters of each specimen were also measured after TU-ESD (Fig. 1G). The surface areas of the ESD and the ulcers were calculated using the mathematic formula *πr*^2^, where

$$r\left( {\text{radius}} \right) = \left( {{\text{Long diameter of specimen}}/ 2+ {\text{ short diameter of specimen}}/ 2} \right)/ 2.$$

All in vitro and in vivo animal studies were performed by experienced medical doctors. The endoscopist, Prof. Chang Yoon Ha, had experience in more than 2000 cases of gastric ESD, and the surgeon, Prof. Sang-Ho Jeong, had experience in 300 cases of laparoscopic gastrectomy with LND at the time of these animal experiments.

### 2. In vivo survival study of TU-ESD with perigastric LND

The protocol for the animal experiments was approved by the Konkuk University Institution Animal Care and Use Committee (KU14162). All applicable international and institutional guidelines for the care and use of animals were followed. The TU-ESD experiments were performed using nine female White Landrace pigs (approximately 40 kg each) between 31 January 2015 and 14 February 2015.

#### Step 1: Animal preparation

The pigs were admitted to the laboratory 1 week before the operation and allowed access to water but not food for 24 h before the day of the TU-ESD experiments. An intramuscular injection of tiletamine/zolazepam (Zoletil, 6 mg/kg) and xylazine (Rompun, 2 mg/kg) served as a preanesthetic. All procedures were performed under general anesthesia with 1.5% to 2% enflurane with 7.0-mm endotracheal intubation. Intravenous access was established via the marginal ear vein. The heart rate, respiration rate, oxygen and carbon dioxide saturation, body temperature, and continuous noninvasive arterial pressure were monitored using the ear artery during the animal experiments.

#### Step 2: Creation of pneumoperitoneum and liver retraction

The anesthetized pig was placed in the supine position. The upper abdomen and the perineal area were disinfected. First, an incision was made in the umbilicus. Pneumoperitoneum was created with the Hasson open technique (approximately 2 cm). After the OCTO Port (OT301; Dalim Surg Net, Seoul, Republic of Korea) was inserted, a laparoscopic 5-mm, 30° telescope was inserted into one of the ports. The cavity was insufflated with CO_2_ (6–8 mmHg of CO_2_ pressure) through the OCTO Port. A laparoflator was used to control the pressure. Bladder catheterization was performed with a feeding tube during the operation. The multilobulated liver was retracted via previously described methods, and temporary duodenal clamping was performed with a laparoscopic bulldog clip [17].

#### Step 3: Creation of the transumbilical route

**Fig. 2:**
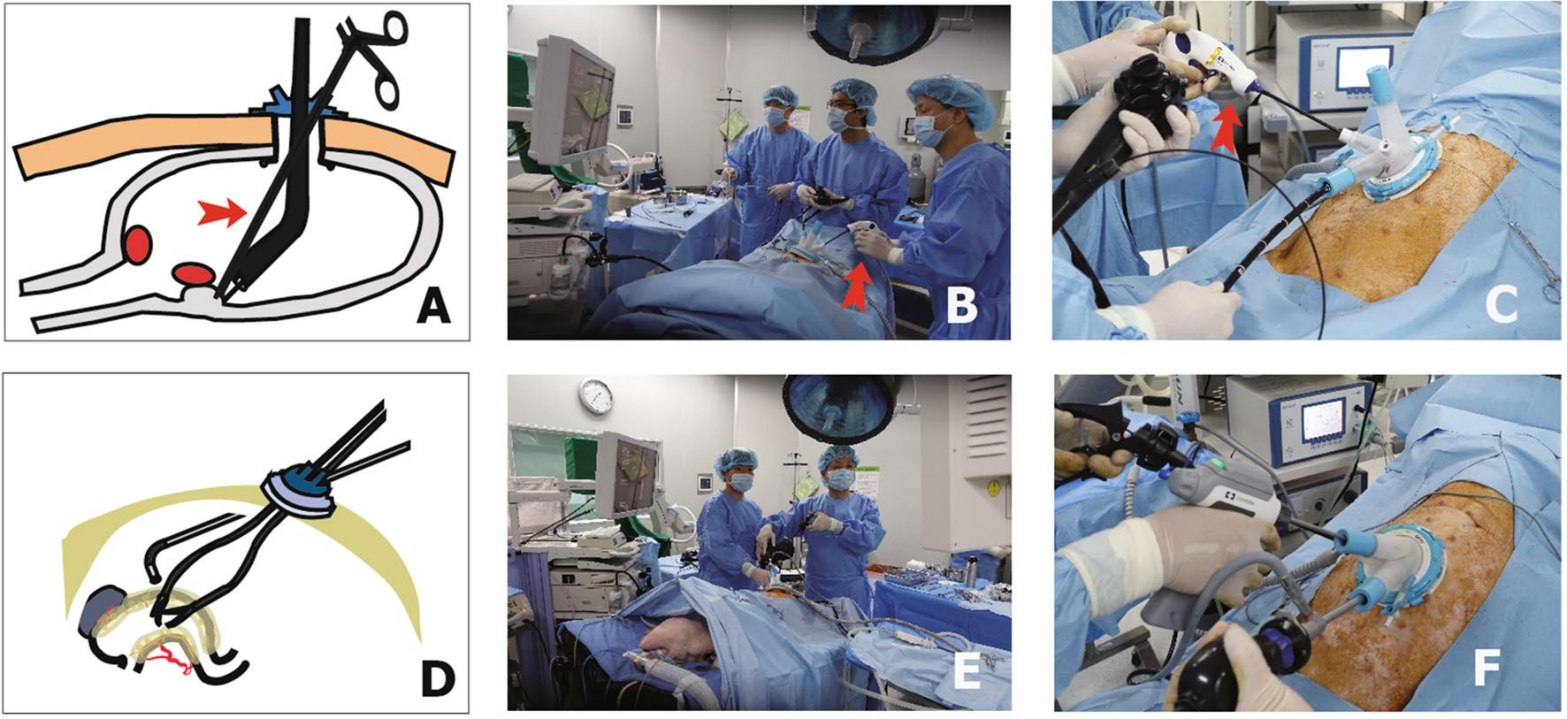
In vivo survival study of TU-ESD performed with perigastric LN dissection. Schematic figure of the TU-ESD procedure used in the in vitro porcine model (**B**). We performed the ESD procedure using an endoscopist with a laparoscopic grasper assist (red arrow, **A**–**C**). After the ESD procedure, we closed the gastrostomy site and re-created a pneumoperitoneum for one-basin LN dissection with a single-port procedure (**D**–**F**)

Under pneumoperitoneum, a gastrostomy site corresponding to the greater curvature side of the antrum AW in humans was selected. The stomach was retracted, and gastrostomy was performed after the OCTO Port was removed. The OCTO Port was inserted through the gastrostomy site to create pneumoperitoneum (Fig. 2A). The cavity was then insufflated with CO_2_ (6 mmHg of CO_2_ pressure) through the OCTO Port.

#### Step 4: TU-ESD

ESD was performed at the AW (*n* = 3), PW (*n* = 3), and LC (*n* = 3) sites of the upper gastric body via the TU route with a single-port L-LND (Fig. 2B, C). ESD was performed using white-light endoscopy and chromoendoscopy with an indigo carmine solution (GIF-H260; Olympus Optical Co., Ltd., Tokyo, Japan).

Marks were made around the lesions with a dual knife, and the mucosa surrounding the lesion was precut. The connective tissue of the submucosa was dissected with an IT-2 knife, and the specimen was retracted using a laparoscopic grasper (Endo Grasp, 5 mm; US Surgical, Norwalk, CT, USA) that was inserted through the OCTO Port (Arrow, Fig. 2A–C). The operation time, tissue weight, specimen diameter, and intraoperative complications were recorded. Finally, the ESD site was examined, and an air-leak test was performed using a laparoscopic instrument. The long and short diameters of each specimen were recorded after TU-ESD to calculate the surface area.

#### Step 5: Gastrostomy repair and pneumoperitoneum creation

After the ESD procedure, the OCTO Port was removed from the gastrostomy site, which was repaired using extracorporeal continuous Polysorb 2-0 sutures. After the area was cleansed with normal saline, the OCTO Port was reinserted to create pneumoperitoneum (Fig. 2D).

#### Step 6: Perigastric single-port L-LND

We ligated the Left gastric vessels and then dissected the regions of the perigastric LNs that corresponded to the LC areas (LNs 1 and 3) in humans using SonoSurg and double-bent instruments (Johan grasping forceps, Maryland dissecting forceps; Olympus, Hamburg, Germany) (Fig. 2E, F). During the operation, the overall completion rate, specimen weight, and operation time were recorded.

#### Step 7: Postoperative care in the TU-ESD group

The pigs were not orally fed for 1 day, after which they were fed soft food for 2 days and then transferred to a solid diet. The pigs were monitored daily in terms of the following: diet, vomiting, defecation, and anal temperature. An intramuscular injection of cefazoline (25 mg/kg/day) was administered to prevent infection from the day of the surgery to postoperative day 5. An intramuscular injection of tramadol (2 mg/kg/day) was administered for pain control from the day of the surgery to postoperative day 3.

**Fig. 3:**
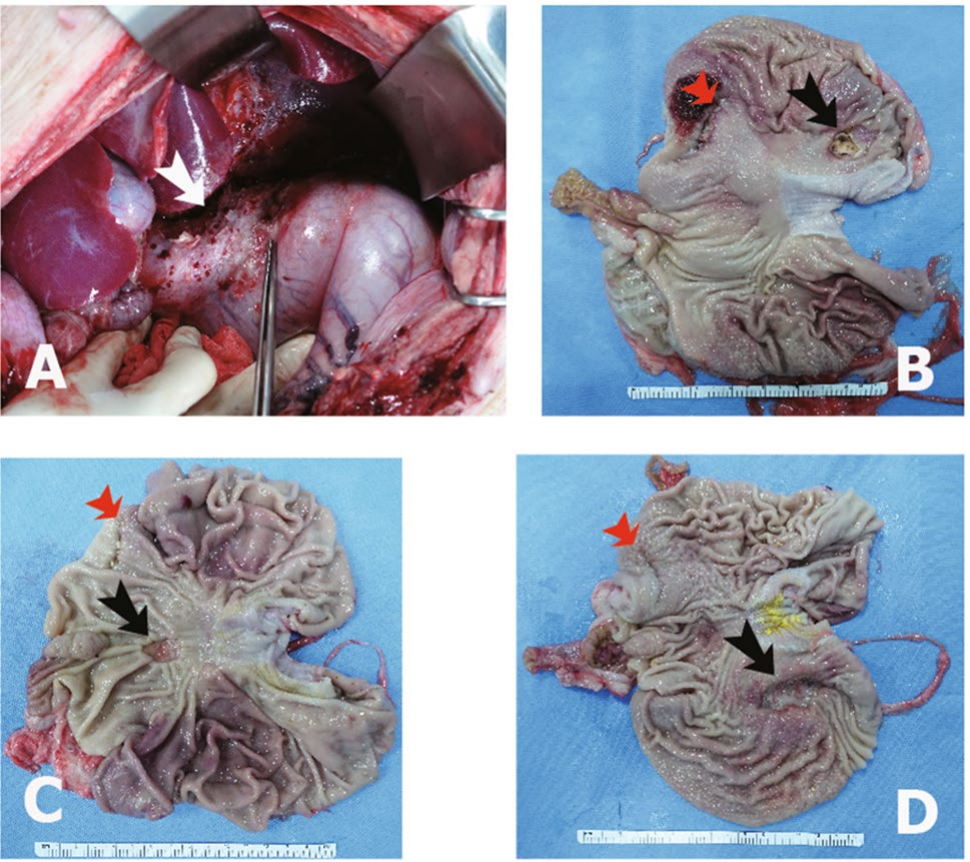
Postoperative sacrifices and examinations, including full laparotomy, were performed on postoperative day 7. We checked the animals for postoperative complications (perforation, bleeding, and abscess formation) and the completeness of single-basin LN dissection (white arrow, LN dissection area) (**A**). After the stomach was retrieved, we checked the size of the gastric healing ulcer and the completeness of endoscopic submucosal dissection (ESD) in the anterior wall group (**B**), the lesser curvature group (**C**), and the posterior wall group (**D**). The black arrow indicates the ulcer area after ESD, and the red arrow indicates the previous gastrostomy site

Seven days after the operation, the pigs were sacrificed and examined by full laparotomy for postoperative complications (perforation, bleeding, and abscess formation) and the completeness of the SBLND (Fig. 3A). After the stomach was retrieved, the size of the healing gastric ulcer was measured along with completeness of the ESD in the AW, LC, and PW (Fig. 3B–D).

## Results

### 1. In an in vitro feasibility study, the TU-ESD operation time was shorter in the PW group than in the LC group

**Fig. 4:**
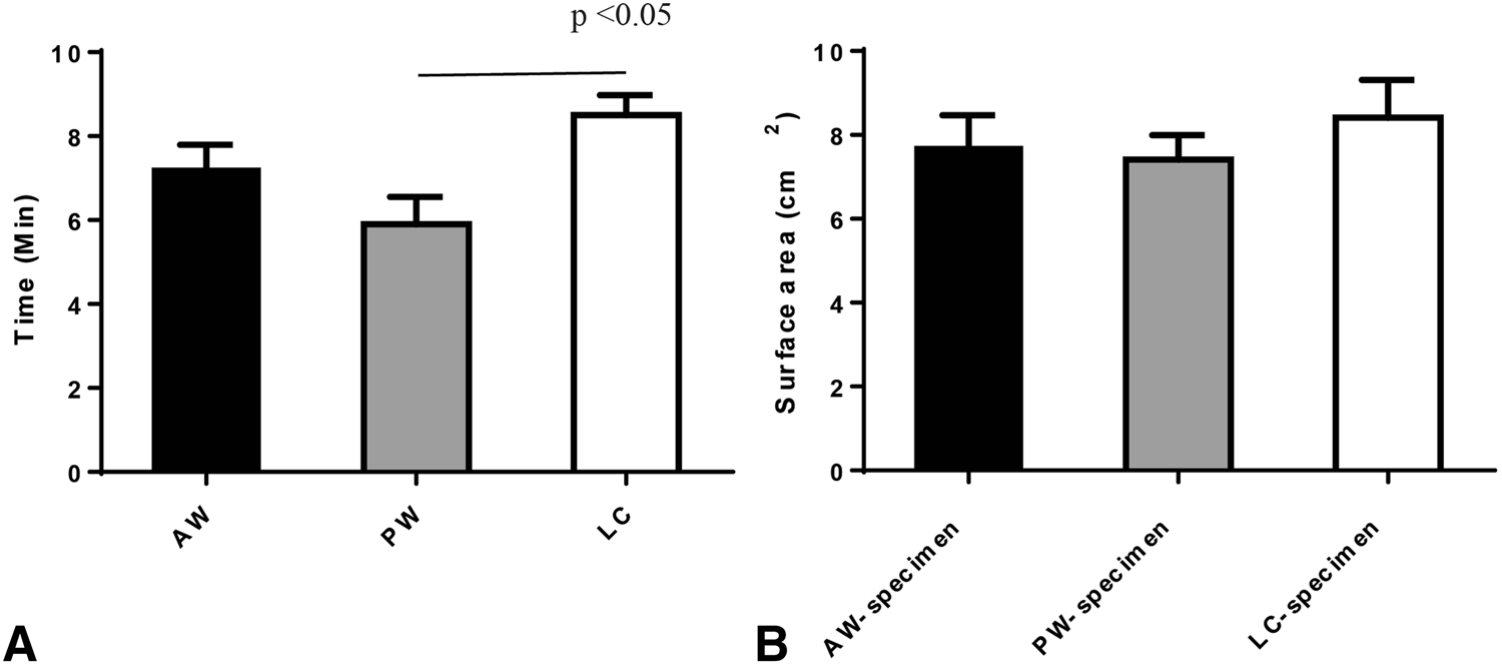
Mean operation times of the TU-ESD procedures (**A**) and mean surface area of the specimens (**B**) according to the circumferential locations used in the in vitro study. *AW* anterior wall group, *PW* posterior wall group, *LC* lesser curvature group

In vitro, the mean TU-ESD operation time for all 10 cases (AW + PW + LC) was 7.2 ± 2.0 min. The TU-ESD time was significantly shorter in the PW group (5.9 ± 2.0 min) than in the LC group (8.5 ± 1.5 min) (*p *< 0.05) (Fig. 4A). The mean times for precut and subcutaneous dissection were 2.7 and 3.2 min, respectively, in the PW group; 3 and 4.2 min, respectively, in the AW group; and 3.1 and 5.4 min, respectively, in the LC group. The mean surface areas of the ESD specimens in the PW, AW, and LC groups were 7.4, 7.6, and 8.4 cm^2^, respectively (*p* = 0.57) (Fig. 4B).

### 2. Surgical results of TU-ESD with SBLND in the survival study

The surgical outcomes of TU-ESD with SBLND are presented in Table 1. TU-ESD with SBLND was completed in all nine pigs without any complications. There were no accidents after the operation and no cases of delayed perforation or bleeding. Secure healing ulcers were found in all pigs 7 days after the operation.

**Table 1:** Surgical outcomes of TU-ESD with one basin dissection

	Value
Complete resection	9/9 (100%)
ESD operation time	8.8 ± 4.3 min
LN dissection time	14.0 ± 2.3 min
ESD specimen weight	1.16 ± 0.43 g
LN weight	4.61 ± 1.94 g
ESD specimen area	8.1 ± 1.9 cm^2^
Ulcere surface area	5.2 ± 3.5 cm^2^
Intraoperative complication	0%
Postoperative complication	0%

*TU-ESD* trans-umbilical endoscopic submucosal dissection, *ESD* endoscopic submucosal dissection, *LN* lymph node

### 3. In the survival study, after 7 days, the ESD ulcers had decreased in size and were healing

**Fig. 5:**
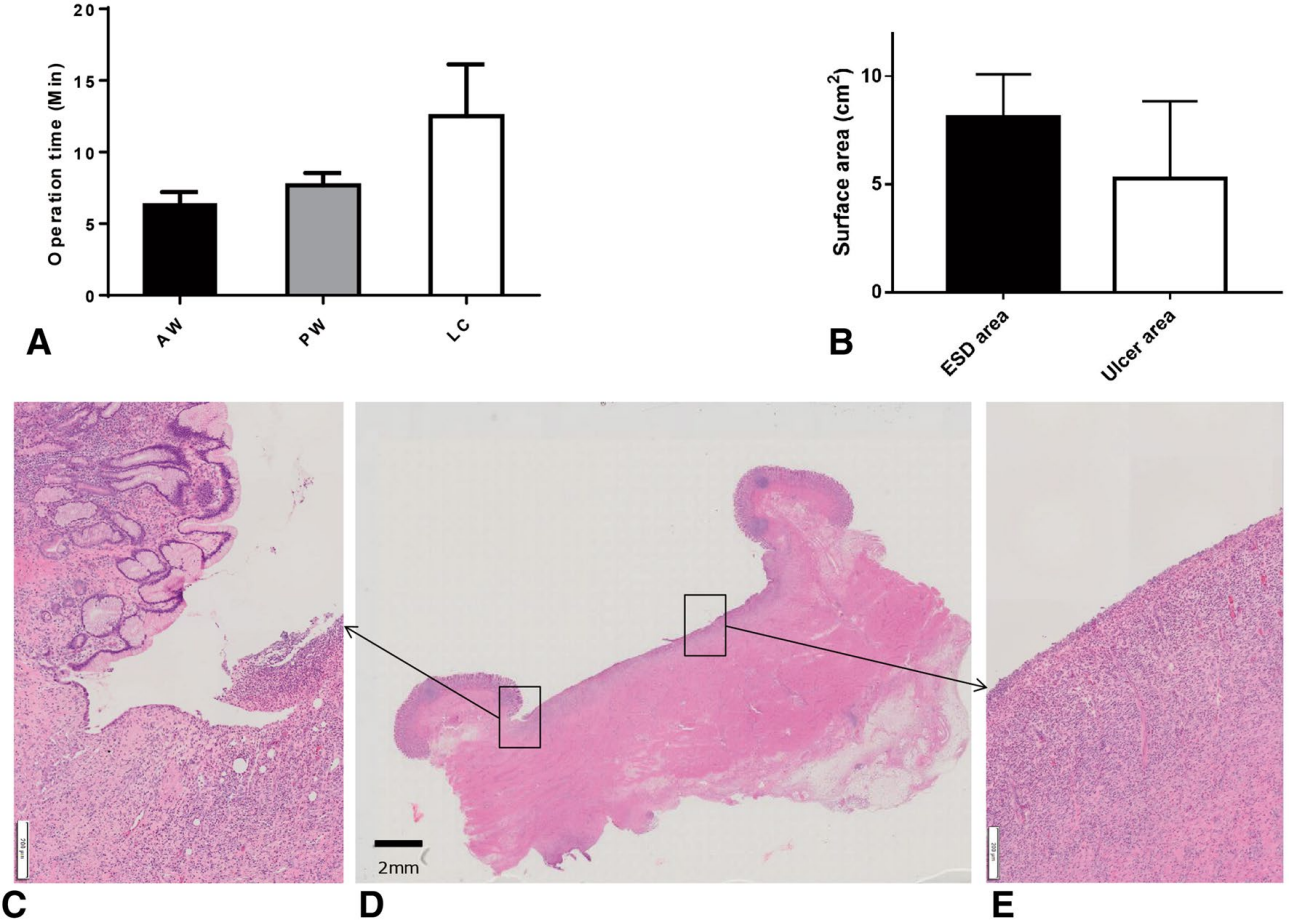
We compared the mean operation times achieved in TU-ESD among the 3 groups (AW, PW, and LC) (**A**). We compared the mean surface areas of the ESD area and the ulcer area (**B**). On microscopy, we found that epithelialization had occurred in the ulcer margin and that there were signs of gastric ulcer healing (**D**, magnified view **C**, **E**)

The mean TU-ESD operation time was 8.8 ± 4.3 min, and the mean L-LND dissection time was 14.0 ± 2.3 min. By circumferential location, the mean operation time, including both TU-ESD and simultaneous L-LND, was shortest for the AW site (6.3 min), followed by the PW site (7.6 min) and the LC site (12.5 min) (Fig. 5A, *p* = 0.60).

The mean surface area of the specimens collected immediately after the TU-ESD procedure was 8.1 ± 1.9 cm^2^, and the mean size of the ulcer was 5.2 ± 3.5 cm^2^ after 7 days. Ulcer size was approximately 36% smaller than the ESD operation site area (*p *= 0.05) (Fig. 5B).

Microscopy showed that epithelialization at the ulcer margin and signs of gastric ulcer healing, indicating that recovery had begun after TU-ESD with SBLND (Fig. 5D, magnified view C, E).

## Discussion

In our previous study, we found that performing ESD via a trans-umbilical route was feasible, had a shorter operation time, and had a lower complication rate than was found for ESD performed in the upper gastric body via a trans-oral route. The most important advantage of trans-umbilical ESD is the possibility that counter-retraction could be used as an assist via a trans-oral endoscope or conventional laparoscopic instruments during the operation [16]. However, delayed gastric perforation occurred in 30% of cases (3/10) treated simultaneously with TU-ESD with perigastric 2-basin L-LND [16]. Therefore, we performed this study to confirm the safety of and that healing occurred successfully after single-basin L-LND. Additionally, we identified suitable circumferential locations for TU-ESD in the upper gastric body. As a result, we found that performing TU-ESD with single-basin L-LND resulted in successful ulcer healing as determined by gross and pathological examinations. We found that the operation time was significantly shorter in the PW group than in the LC group, indicating that the PW could be a more convenient site for performing TU-ESD with single-basin L-LDN. To the best of our knowledge, our study presents unique in vivo results showing the delayed gastric perforation rate after ESD with LND.

Tumor location can influence the completeness and safety of ESD for EGC. Lesions in the PW and upper third of the stomach reportedly have significantly higher rates of incomplete resection and longer procedure times than are achieved in lesions in other areas of the stomach [5]. Hence, endoscopists need to take care when applying advanced endoscopic techniques to perform ESD for lesions in these locations to improve clinical outcomes and minimize the rate of serious complications. TU-ESD may provide an advantage by producing less stricture after the procedure in cardiac cancer than is achieved by wedge resection or endoscopic full-thickness resection (EFTR). The most important advantage of TU-ESD is the possibility using countertraction assistance during the operation; additionally, this approach may reduce operation times and the complication rate.

Among the possible complications, delayed gastric perforation can occur even in cases in which no visible perforations are observed during the ESD procedure or in those with eminent clinical symptoms, suggesting that perforation occurred just after the procedure. The two types of gastric perforation after ESD can be defined according to the postprocedure time of onset: intraoperative and delayed perforation [18]. The delayed-onset type is usually found after a short time and shows no evidence of free air on abdominal radiography during or immediately after the ESD procedure. Delayed gastric perforation mostly occurs within 7 days after ESD [19]. Therefore, in our protocol, we assessed the healing status of the ESD sites in a full laparotomy at 7 days after the operation.

Although the incidence of delayed perforation after gastric ESD is quite low (0.1% to 0.45%), it is essential to be extremely careful because in many cases, perforation requires interventions, including emergent surgery [18–20]. Delayed perforation might be related to necrosis of the gastric muscle and serosal layer caused by insufficient blood circulation around the dissected site. Therefore, in cases of delayed perforation after ESD, treatment with emergency surgery should be considered instead of conservative management. Some reports have evaluated delayed perforation after gastric ESD; however, few published reports have thoroughly investigated the outcomes of delayed perforation after ESD with synchronous LND based on data from a large series of consecutive EGC patients. Kilgore et al. reported that in an animal study, they observed no complications after subtotal gastrectomy with a dual supply from the left gastric artery and vasa brevia from the splenic artery; however, a 20% perforation rate (5/25) was observed when 1 supply was applied, and an 83% perforation rate (10/12) was observed in cases completed without a left gastric artery or vasa brevia supply [21]. In our previous study, we found that the delayed perforation rate was 30% in two-basin LN dissections [16] when we ligated left gastric vessels, Lt gastroepiploic vessels, and short gastric vessels. In this study, we ligated only left gastric vessels in one-basin dissection procedures, and we preserved the left gastroepiploic and short gastric vessels in one-basin procedures but not two-basin procedures. This may be because the blood supply at the ESD site is relatively more sufficient than that of the two-basin L-LDN site used in our previous animal experiment.

Postoperative ischemia or leakage occurs via the same mechanism involved in delayed gastric perforation. The sacrifice of vessels in both the greater curvature and the LC territories increases the chance of ischemia. Cho et al. reported a case of postoperative ischemia in which the lesion was a 2-cm tumor with submucosal invasion in the midbody AW. They performed EFTR with sentinel LND and saved the left gastric artery and left gastroepiploic artery, but the stomach showed ischemia after EFTR with LND. Thus, they performed an additional gastrectomy. They also reported a case in which leakage occurred after treatment for cancer in the midbody of the LC, and we believe that this leakage could also have been caused by postoperative ischemia [22]. Conversion to gastrectomy is the safest method to avoid an ischemic event. To save the stomach without gastrectomy, vessel-preserving basin dissection with concurrent ESD could be considered an alternative treatment modality.

There are some limitations to the present study. The sample size for the in vivo experiments was small, and the epigastric vascular anatomy of pigs may be different from that of humans. Additionally, in the in vitro study performed using an EASIE-R tray, we did not take into consideration the various parameters involved in the actual operation, such as bleeding. Nevertheless, to the best of our knowledge, this is the first study to present a quantification of the healing process that occurs in ESD with LND in which similar efficacy was achieved both in vitro and in vivo. Another merit of our procedure is that it is easy to use because all of our procedures involve the use of conventional endoscopic and laparoscopic devices.

In conclusion, we found that TU-ESD with synchronous single-basin L-LND is a feasible and safe method for the removal of gastric lesions. Consequently, we plan to use these operation methods in human trials. The PW of the upper gastric body could be the most suitable location for performing TU-ESD, perhaps because of the difference in subcutaneous dissection times.
